# Supplementary material for: Exploring the effects of habitat management on grassland biodiversity: A case study from northern Serbia
Source: PLoS One. 2024 Mar 28;19(3):e0301391. doi: 10.1371/journal.pone.0301391 (PMC10977728; doi:10.1371/journal.pone.0301391)
Supplement: S1 Table — (PDF) [file pone.0301391.s001.pdf]

Supporting information captions

S1 Table. List of recorded species at Subotica sand (Serbia)

Plant species

1. *Achillea aspleniifolia* Vent.
2. *Achillea setacea* Waldst. & Kit.
3. *Adonis vernalis* L.
4. *Alopecurus pratensis* L.
5. *Alyssum alyssoides* (L.) L.
6. *Ambrosia artemisiifolia* L.
7. *Anchusa officinalis* L.
8. *Anthemis ruthenica* M.Bieb.
9. *Anthoxanthum odoratum* L.
10. *Apera spica-venti* (L.) P.Beauv.
11. *Arabidopsis thaliana* (L.) Heynh.
12. *Arabis hirsuta* (L.) Scop.
13. *Arabis recta* Vill.
14. *Arabis sagittata* (Bertol.) DC.
15. *Arenaria serpyllifolia* L.
16. *Aristolochia clematitis* L.
17. *Arrhenatherum elatius* (L.) P.Beauv. ex J.Presl & C.Presl.
18. *Asclepias syriaca* L.
19. *Asparagus officinalis* L.
20. *Asperula cynanchica* L.
21. *Briza media* L.
22. *Bromus inermis* Leyss.
23. *Bromus hordeaceus* L.
24. *Bromus ramosus* Huds.
25. *Bromus squarrosus* L.
26. *Bromus sterilis* L.
27. Bromus tectorum L.
28. *Calamagrostis epigejos* (L.) Roth
29. *Calepina irregularis* (Asso) Thell.
30. *Capsella bursa-pastoris* (L.) Medik.
31. *Carduus acanthoides* Pall. ex M.Bieb.
32. *Carduus nutans* L.
33. *Carex caryophyllea* Latourr.
34. *Carex hirta* L.
35. *Centaurea jacea* subsp. *banatica* (Roch.) Hayek
36. *Centaurea jacea* L.
37. *Cerastium brachypetalum* Desp. ex Pers.
38. *Cerastium glomeratum* Thuill.
39. *Cirsium arvense* (L.) Scop.
40. *Cirsium vulgare* (Savi) Ten.
41. *Consolida regalis* Gray
42. *Convolvulus arvensis* L.
43. *Cornus mas* L.
44. *Coronilla varia* L.
45. *Crataegus monogyna* Jacq.
46. *Crepis setosa* Haller f.
47. *Cruciata pedemontana* (Bellardi) Ehrend.
48. *Dactylis glomerata* L.
49. *Descurainia sophia* (L.) Webb ex Prantl
50. *Dianthus giganteus* d'Urv. subsp. *banaticus* (Heuff.) Tutin
51. *Dianthus giganteiformis* (Borbás) Heinr.Braun subsp. *pontederae* (A.Kern.) Soó
52. *Digitaria filiformis* (L.) Koeler
53. *Draba nemorosa* L.
54. *Elymus repens* (L.) Gould
55. *Equisetum arvense* L.
56. *Equisetum ramosissimum* Desf.
57. *Erigeron annuus* (L.) Pers.
58. *Erigeron canadensis* L.
59. *Erodium cicutarium* (L.) L'Hér.
60. *Eryngium campestre* L.
61. *Erysimum diffusum* Ehrh.
62. *Euphorbia cyparissias* L.
63. *Euphorbia virgata* Waldst. & Kit.
64. *Falcaria vulgaris* Bernh.
65. *Festuca ovina* L.
66. *Festuca rupicola* Heuff.
67. *Festuca vaginata* Waldst. & Kit. ex Willd.
68. *Festuca valesiaca* Schleich. ex Gaudin
69. *Filipendula ulmaria* (L.) Maxim.

70. *Fraxinus ornus* L.
71. *Galium austriacum* Jacq.
72. *Galium verum* L.
73. *Galium austriacum* Jacq.
74. *Genista tinctoria* L.
75. *Geranium molle* L.
76. *Hieracium echioides* Lumn.
77. *Hordeum jubatum* L.
78. Jacobaea vulgaris Gaertn.
79. *Knautia arvensis* (L.) Coult.
80. *Knautia drymeia* Heuff.
81. *Lamium amplexicaule* L.
82. *Lamium purpureum* L.
83. *Lathyrus aphaca* L.
84. *Lathyrus pratensis* L.
85. *Ligustrum vulgare* L.
86. *Linum austriacum* L.
87. *Lithospermum arvense* L.
88. *Lolium perenne* L.
89. *Lotus corniculatus* L.
90. *Matricaria inodora* L.
91. *Medicago minima* (L.) L.
92. *Melica transsilvanica* Schur
93. *Minuartia glomerata* (M.Bieb.) Degen
94. *Molinia caerulea* (L.) Moench
95. *Muscari neglectum* Guss. ex Ten.
96. *Myosotis arvensis* (L.) Hill
97. *Onobrychis arenaria* (Kit.) DC.
98. *Ononis spinosa* L.
99. *Papaver rhoeas* L.
100. *Papaver somniferum* L.
101. *Phleum phleoides* (L.) H.Karst.
102. *Phleum pratense* L.
103. *Phragmites australis* (Cav.) Trin. ex Steud.
104. *Plantago lanceolata* L.
105. *Plantago major* L.
106. *Poa pratensis* L.
107. *Podospermum canum* C.A.Mey.
108. *Polygala comosa* Schkuhr
109. *Potentilla incana* P.Gaertn., B.Mey. & Scherb.
110. *Prunus spinosa* L.
111. *Ranunculus polyanthemos* L.
112. *Rhinanthus borbasii* (Dörfl.) Soó
113. *Rosa canina* L.
114. *Rubus* sp.
115. *Rumex acetosa* L.
116. *Rumex acetosella* L.
117. *Salix* sp.
118. *Salvia austriaca* Jacq.
119. *Salvia nemorosa* L.
120. *Salvia pratensis* L.
121. *Sanguisorba minor* Scop.
122. *Sanguisorba officinalis* L.
123. *Saxifraga tridactylites* L.
124. *Scabiosa leucocephala* L.
125. *Scabiosa leucocephala*
126. *Scirpoides holoschoenus* (L.) Soják
127. *Schoenoplectiella supina* (L.) Lye
128. *Secale sylvestre* Host
129. *Senecio squalidus* subsp. *rupestris* (Waldst. & Kit.) Greuter)
130. *Sherardia arvensis* L.
131. *Silene latifolia* subsp. *alba* (Mill.) Greuter & Burdet
132. *Silene conica* L.
133. *Silene dioica* (L.) Clairv.
134. *Silene vulgaris* (Moench) Garcke
135. *Sisymbrium orientale* L.
136. *Solidago gigantea* Aiton
137. *Solidago virgaurea* L.
138. *Sonchus asper* (L.) Hill
139. *Stellaria pallida* (Dumort.) Crép.
140. *Stipa pennata* L.
141. Tanacetum vulgare L.
142. *Taraxacum officinale* (L.) Weber ex F.H.Wigg.
143. Teucrium chamaedrys L.
144. *Thalictrum minus* L.
145. *Thlaspi perfoliatum* L.
146. *Thymus serpyllum* L.
147. *Trifolium arvense* L.
148. *Trifolium campestre* Schreb.
149. *Trifolium diffusum* Ehrh.
150. *Trifolium montanum* L.
151. *Valerianella locusta* (L.) Laterr.
152. *Verbascum blattaria* L.
153. *Verbascum officinarum* Crantz
154. *Verbascum phoeniceum* L.
155. *Veronica arvensis* L.

156. *Veronica austriaca* L.
157. *Veronica verna* L.
158. *Veronica vindobonensis* (M.A.Fisch.) M.A.Fisch.
159. *Vicia sativa* subsp. *nigra* (L.) Ehrh.
160. *Vicia cracca* L.
161. *Vicia hirsuta* (L.) Gray
162. *Vicia sativa* L. subsp. sativa
163. *Viola arvensis* Murray
164. *Viola kitaibeliana* Schult.

<u>Pollinators</u>

Bees

1. *Andrena taraxaci* Giraud, 1861
2. *Andrena florea* Fabricius, 1793
3. *Amegilla quadrifasciata* de Villers, 1789
4. *Bombus argillaceus* (Scopoli, 1763)
5. *Bombus terrestris* Linnaeus, 1758
6. *Bombus lapidarius* Linnaeus, 1758
7. *Bombus sylvarum* Linnaeus, 1761
8. *Bombus humilis* Illiger, 1806
9. *Eucera interrupta* Bär, 1850
10. *Eucera nigrescens* Pérez, 1879
11. *Eucera clypeata* Erichson, 1835
12. *Xylocopa violacea* Linnaeus, 1758
13. *Xylocopa valga* Gerstäcker, 1872
14. *Halictus simplex* Blüthgen, 1923
15. *Osmia bicolor* Schrank, 1781
16. *Coelioxys inermis* Kirby, 1802
17. *Megachile ericetorum* Lepeletier, 1841
18. *Apis mellifera* Linnaeus, 1758

Hoverflies

1. *Cheilosia aerea* Dufour, 1848
2. *Chrysotoxum cautum* (Harris, 1776)
3. *Chrysotoxum festivum* (Linnaeus, 1758)
4. *Epistrophe eligans* (Harris, 1780)
5. *Eristalis arbustorum* (Linnaeus, 1758)
6. *Eristalis tenax* (Linnaeus, 1758)
7. *Helophilus trivittatus* (Fabricius, 1805)
8. *Melanostoma mellinum* (Linnaeus, 1758)
9. *Merodon nigritarsis* Rondani, 1845
10. *Paragus haemorrhous* Meigen, 1822
11. *Pipizella viduata* (Linnaeus, 1758)
12. *Sphaerophoria scripta* (Linnaeus, 1758)
13. *Syritta pipiens* (Linnaeus, 1758)

Birds

1. *Aegithalos caudatus* (Linnaeus, 1758)
2. *Acrocephalus palustris* (Bechstein, 1798)
3. *Alauda arvensis* Linnaeus, 1758
4. *Anas platyrhynchos* Linnaeus, 1758
5. *Anthus campestris* (Linnaeus, 1758)
6. *Anthus trivialis* (Linnaeus, 1758)
7. *Ardea cinerea* Linnaeus, 1758
8. *Buteo buteo* (Linnaeus, 1758)
9. *Linaria cannabina* (Linnaeus, 1758)
10. *Carduelis carduelis* (Linnaeus, 1758)
11. *Chloris chloris* (Linnaeus, 1758)
12. *Certhia brachydactyla* Brehm, 1820
13. *Circus aeruginosus* (Linnaeus, 1758)
14. *Circus pygargus* (Linnaeus, 1758)
15. *Coccothraustes coccothraustes* (Linnaeus, 1758)
16. *Columba palumbus* Linnaeus, 1758
17. *Corvus corax* Linnaeus, 1758
18. *Coracias garrulus* Linnaeus, 1758
19. *Corvus corone* Linnaeus, 1758
20. *Coturnix coturnix* (Linnaeus, 1758)
21. *Cuculus canorus* Linnaeus, 1758
22. *Dendrocopos major* (Linnaeus, 1758)
23. *Leiopicus medius* (Linnaeus, 1758)
24. *Dryocopus martius* (Linnaeus, 1758)
25. *Emberiza calandra* Linnaeus, 1758
26. *Erithacus rubecula* (Linnaeus, 1758)
27. *Falco tinnunculus* Linnaeus, 1758
28. *Fringilla coelebs* Linnaeus, 1758
29. *Garrulus glandarius* (Linnaeus, 1758)
30. *Hippolais icterina* (Vieillot, 1817)
31. *Hirundo rustica* Linnaeus, 1758
32. *Jynx torquilla* Linnaeus, 1758
33. *Lanius collurio* Linnaeus, 1758
34. *Lanius* minor Gmelin, 1788
35. *Lullula arborea* (Linnaeus, 1758)
36. *Luscinia megarhynchos* (Brehm, 1831)
37. *Merops apiaster* Linnaeus, 1758
38. *Motacilla* alba Linnaeus, 1758
39. *Muscicapa striata* (Pallas, 1764)
40. *Oriolus oriolus* (Linnaeus, 1758)
41. *Cyanistes caeruleus* (Linnaeus, 1758)
42. *Parus major* Linnaeus, 1758
43. *Passer montanus* (Linnaeus, 1758)
44. *Phasianus colchicus* (Linnaeus, 1758)
45. *Phoenicurus ochruros* (Gmelin, 1774)
46. *Phylloscopus collybita* (Vieillot, 1817)
47. *Phylloscopus sibilatrix* (Bechstein, 1793)
48. *Pica pica* (Linnaeus, 1758)
49. *Picus viridis* Linnaeus, 1758
50. *Saxicola torquatus* (Linnaeus, 1766)
51. *Sitta europaea* Linnaeus, 1758
52. *Streptopelia turtur* (Linnaeus, 1758)
53. *Sturnus vulgaris* Linnaeus, 1758
54. *Sylvia atricapilla* (Linnaeus, 1758)
55. *Curruca communis* (Latham, 1787)
56. *Curruca curruca* (Linnaeus, 1758)
57. *Turdus merula* Linnaeus, 1758
58. *Turdus philomelos* Brehm, 1831
59. *Turdus viscivorus* Linnaeus, 1758
60. *Upupa epops* Linnaeus, 1758
61. *Vanellus vanellus* (Linnaeus, 1758)
