# Supplementary material for: Exploring the effects of habitat management on grassland biodiversity: A case study from northern Serbia
Source: PLoS One. 2024 Mar 28;19(3):e0301391. doi: 10.1371/journal.pone.0301391 (PMC10977728; doi:10.1371/journal.pone.0301391)
Supplement: Attachment — Submitted filename: my comments.docx [file pone.0301391.s002.docx]

**Line-by-line comments:**

Line 86: change “forging” to “foraging”

Line 124: I’m not sure what is meant by “degradation stadium”

Figure 1: It’s difficult to see the labels in the figure that show where the 3 different treatments are located. Can the figure be edited to make these more readable?

Line 167: The “nearest three” what? Nearest three other sites?

Lines 162-170: I would like to see more detail here because I don’t think these methods are clear as currently written. How were the habitats characterized? It sounds like there is some focal habitat that was characterized, as well as a 150 m buffer zone that presumably was also characterized by habitat type. Did the measures of tree/shrub cover determine the characterization of habitat types? How large is the focal study area at each of the sites?

Line 183: I’m not familiar with “social behavior types” for plants. What is this?

Lines 190-192: How were the bee identifications conducted, and by whom? The person who did the work should be named, and the taxonomic keys that were used to identify bees should be named and cited. (See Packer et al. 2018. Validating taxonomic identifications in entomological research. *Insect Conservation and Diversity* 11: 1-12 for more information.)

Lines 234-236: It would be great to see a little more detail about what decision trees are in a general sense and what benefit the authors think they bring to this research.

Lines 260-262: It is not easy to see this point in Figure 2, especially for vegetation height (Fig. 2A). Can the authors revise this sentence to better summarize the figure?

Figure 2: It’s very difficult to tell which parts of the figure the uppercase letters A, B, and C are referring to. Can the authors make this clearer? Are the uppercase letters just pointing out that management type was significant across sites in their ANOVA analysis?

Lines 282-283: Some of these numbers don’t make sense: “four species with >10 and 103 individuals”
