# Supplementary material for: Exploring the effects of habitat management on grassland biodiversity: A case study from northern Serbia
Source: PLoS One. 2024 Mar 28;19(3):e0301391. doi: 10.1371/journal.pone.0301391 (PMC10977728; doi:10.1371/journal.pone.0301391)
Supplement: Attachment — Submitted filename: Response to reviewer comments_Milic et al..docx [file pone.0301391.s003.docx]

Dear Editor,

I have received the reports of you and two reviewers on the manuscript “**Exploring the effects of habitat management on grassland biodiversity: A case study from northern Serbia**”. I am glad to see that manuscript could be reconsidered for publication if revisions were prepared. Following reviewer’s comments strictly, we made certain changes in the manuscript.

The funders had no role in study design, data collection and analysis, decision to publish, or preparation of the manuscript.

Land-use data for Figure 1. were obtained from Copernicus Global Land Service (<https://land.copernicus.eu/>). According to 4.1 Terms of Use of Copernicus Global Land Service: The product(s) described in this document is/are created in the frame of the Copernicus programme of the European Union by the European Environment Agency (product custodian) and is/are owned by the European Union. The product(s) can be used following Copernicus full free and open data policy, which allows the use of the product(s) also for any commercial purpose. Derived products created by end users from the product(s) described in this document are owned by the end users, who have all intellectual rights to the derived products (https://land.copernicus.eu/en/technical-library/product-user-manual-for-clc-backbone-raster-only/@@download/file). The sources of the layers used for map creation, including cities, are specified in the caption of Figure 1. Furthermore, the map has not been previously copyrighted to our knowledge.

Together with the revised manuscript, we are sending a list of responses to the comments. We have endeavored to respond to all the points raised. As detailed below, we have checked all the general and specific comments provided by the Referees and have made the necessary changes according to their recommendations.

The authors would like to express their appreciation to the reviewers and editors for their help and useful comments.

Sincerely,

Dubravka Milić

**Reviewers' comments:**

**Reviewer #1**: This study focuses on the effects of different grassland management types, specifically grazing and mowing compared to control, on the diversity of three different groups: plants, pollinators, and birds. They show differences in the diversity of these three groups in response to management. In my specific comments on the paper I've highlighted places where I think additional methodological details are needed.

**Line-by-line comments:**

**Reviewer comment:** Line 86: change “forging” to “foraging”

***Milić et al.:*** *corrected. We accept the Reviewer's remark*.

**Reviewer comment:** Line 124: I’m not sure what is meant by “degradation stadium”

***Milić et al.:*** *Pruno spinosae-Crataegetum is degradation stadium of Convallario-Quercetum roboris. We rephrased the sentence to provide a clearer explanation.*

**Reviewer comment:** Figure 1: It’s difficult to see the labels in the figure that show where the 3 different treatments are located. Can the figure be edited to make these more readable?

***Milić et al.:*** *We modified Figure 1 to align with the journal's requirements and enhance its readability.*

**Reviewer comment:** Line 167: The “nearest three” what? Nearest three other sites?

***Milić et al.:*** *It was our mistake, we should have written “the nearest trees”. We have corrected in our manuscript.*

**Reviewer comment:** Lines 162-170: I would like to see more detail here because I don’t think these methods are clear as currently written. How were the habitats characterized? It sounds like there is some focal habitat that was characterized, as well as a 150 m buffer zone that presumably was also characterized by habitat type. Did the measures of tree/shrub cover determine the characterization of habitat types? How large is the focal study area at each of the sites?

***Milić et al.:*** *We accept the Reviewer's remark. Throughout the 'Study Area' section, we have made modifications to the text and added an additional table. In this table, we have categorized the habitat types according to our National classification of Republic of Serbia and mark which habitat type corresponds to NATURA 2000 habitat type. This was done to facilitate the identification of habitat types within the research area. Furthermore, we wrote that each sampling site covering an area of 1 hectare.*

**Reviewer comment:** Line 183: I’m not familiar with “social behavior types” for plants. What is this?

***Milić et al.:*** *Social behavior types (SBT) are defined by the role that a plant species plays within its community. These categories represent how a particular plant is interconnected with its habitat, conveying valuable information about the naturalness of this connection. The presence of these SBTs within a community can provide insights into various aspects, such as the ecological richness of the community, its stability, its natural state, niche occupancy, regenerative potential, and the degree of disturbance, transformation, or deviation from the natural state. According to Borhidi (1995), these categories include specialists (S), generalists (G), disturbance tolerants (DT), natural pioneers (NP), natural competitors (C), indigenous ruderal competitors (RC), and alien competitors and introduced species (AC + I).*

*We have added the paragraph: In addition, in order to gain insights into community dynamics and ecological characteristics, we employed Social Behavior Types (SBT) for all plant species. SBT categorizes plant species based on their roles in the community and provides valuable information about community richness, stability, naturalness, niche occupancy, and response to disturbances or deviations from the natural state [38].”*

**Reviewer comment:** Lines 190-192: How were the bee identifications conducted, and by whom? The person who did the work should be named, and the taxonomic keys that were used to identify bees should be named and cited. (See Packer et al. 2018. Validating taxonomic identifications in entomological research. *Insect Conservation and Diversity* 11: 1-12 for more information.)

***Milić et al.:*** *Thank you for your comment; it was an oversight on our part. The author of this manuscript, Sonja Mudri-Stojnić, was responsible for identifying bee species and Tamara Tot for hoverflies. We have included the taxonomic keys that were used for bee species as well as for hoverflies identification*.

**Reviewer comment:** Lines 234-236: It would be great to see a little more detail about what decision trees are in a general sense and what benefit the authors think they bring to this research.

***Milić et al.:*** *We added a paragraph in section Materials and methods discussing the general concept and benefits of the decision tree.*

**Reviewer comment:** Lines 260-262: It is not easy to see this point in Figure 2, especially for vegetation height (Fig. 2A). Can the authors revise this sentence to better summarize the figure?

***Milić et al.:*** *We accept the Reviewer's remark. We have revised the sentence and also made correction on Figure 2*.

**Reviewer comment:** Figure 2: It’s very difficult to tell which parts of the figure the uppercase letters A, B, and C are referring to. Can the authors make this clearer? Are the uppercase letters just pointing out that management type was significant across sites in their ANOVA analysis?

***Milić et al.:*** *Yes****,*** *uppercase letters indicate significant differences between grazing, mowing and control (different management practice). We also have made clearer Figure 2.*

**Reviewer comment:** Lines 282-283: Some of these numbers don’t make sense: “four species with >10 and 103 individuals”

***Milić et al.:*** *corrected. We have changed the sentence:* ***“****Most of the species had a very low abundance, with 25 species having 10 or fewer individuals, five species having 10 to 30 individuals, and only one species having over 100 individuals.”*

**Reviewer #2**: Exploring the effects of habitat management on grassland biodiversity: A case study from northern Serbia.

This an interesting study presenting the effect of grassland management (mowing and grazing) on different biodiversity taxa. Although the study is valuable, it could be much improved. Some parts of the manuscript are not easy to follow, especially the introduction, due to poor writing. And the discussion section is too lengthy, should be shortened and more focused to the main questions assessed in the study. But the main concern is the sampling design, which is unbalanced (only 1 control site vs. 3 grazing and 3 mowing sites) and this should be acknowledged and dealt with proper statistical analysis in the study.

***Milić et al.****: We made some modifications to the Introduction section and condensed the discussion, aligning it more closely with the questions addressed in the study based on the reviewer's suggestions. Additionally, we included a paragraph detailing the statistical analysis employed to address the issue of imbalance in study sites.*

Minor comments

**Reviewer comment:** L81: I recommend changing the sentence to make it clearer. By “initiatives” you refer to the conservation strategies? Something like “these conservation initiatives cover ecosystem services such as biomass production and…”, as you are referring here to the ecosystem services grasslands provide

***Milić et al.****: We have changed the sentence according Reviewer’s recommendation.*

**Reviewer comment:** L84 To what species you are referring to by “as many species are presently threatened”? Of solitary bees

***Milić et al.****: Yes, we meant on solitary bees. We have changed the sentence to make it clearer.*

**Reviewer comment:** L83-87 the whole sentence would benefit from some rephrasing

***Milić et al.****: We have rephrased whole sentence.*

**Reviewer comment:** L86. Fix typo, forging should be “foraging”

***Milić et al.****: corrected*

**Reviewer comment:** L167: fix typo, nearest tree (NOT three)

***Milić et al.****: corrected*

**Reviewer comment:** L171-175: treatments could be explained in a more structured and clear way, without mixing information

***Milić et al.****: We have restructured and clarified the presentation in this paragraph, separating the information more distinctly.*

**Reviewer comment:** L186-192: no information on time of the day when pollinator sampling was conducted, please add

***Milić et al.****: We added information about the time of day and weather conditions during which the transect walks were conducted.*

**Reviewer comment:** L205: I suggest rephrasing, species diversity was "calculated" instead of interpreted.

***Milić et al.****: corrected*

**Reviewer comment:** L249: In the study area is not needed, I suggest to delete that

***Milić et al.****: corrected*

**Reviewer comment:** L250. Please rephrase. Recorded instead of “noted presence”

***Milić et al.****: corrected*

**Reviewer comment:** L253: Please correct. Unmanaged site, singular as there was only one site.

***Milić et al.****: corrected*

**Reviewer comment:** Table 1. Are those numbers mean % across sites? Please specify. Also, would be better if the description of social behaviour types in table legend text follows the order of appearance of the table, starting with S, D, DT, NP and IAS

***Milić et al.****: We accept the Reviewer's remark. We have changed the title of the table to be more specified and have changed legend text follows the order of appearance of the table.*

**Reviewer comment:** Table 4. Fix typo, distance to nearest TREE

***Milić et al.****: corrected*

**Reviewer comment:** Fig. 3. IT would help to add the full name of management type in the legend in the figure: Control, Grazing, and Mowing. Also Variat should be replaced by Management.

***Milić et al.****: In Figure 3 we have added the full name of the management practice and replaced 'Variat' with 'Management' according Reviewer’s suggestion.*

**Reviewer comment:** Discussion could be shortened in order to improve flow and focus on main messages.

***Milić et al.****: We made the Discussion section shortened, focusing on the main messages in our research. We hope that we have successfully conveyed our key findings and insights.*
